# Metabolomics Reveals *Lysinibacillus capsici* TT41-Induced Metabolic Shifts Enhancing Drought Stress Tolerance in Kimchi Cabbage (*Brassica rapa* L. subsp. *pekinensis*)

**DOI:** 10.3390/metabo14020087

**Published:** 2024-01-25

**Authors:** Tae Jin Kim, Ye Ji Hwang, Young Jin Park, Jong Sung Lee, Jae Kwang Kim, Mi-Hwa Lee

**Affiliations:** 1 Bio-Resource Industrialization Center, Nakdonggang National Institute of Biological Resources, Sangju 37242, Republic of Korea; f91gd@nnibr.re.kr (T.J.K.); hyj86@nnibr.re.kr (Y.J.H.); 2 Division of Life Sciences, College of Life Sciences and Bioengineering, Incheon National University, Incheon 22012, Republic of Korea; pyj2050@inu.ac.kr (Y.J.P.); 202322017@inu.ac.kr (J.S.L.)

**Keywords:** drought stress, abiotic stress, lactic acid, metabolomics, *Brassica rapa* L., cabbage

## Abstract

Climate change has increased variable weather patterns that affect plants. To address these issues, we developed a microbial biocontrol agent against drought stress in kimchi cabbage (*Brassica rapa* L. subsp. *pekinensis*). We selected three bacterial strains (*Leifsonia* sp. CS9, *Bacillus toyonensis* TSJ7, and *Lysinibacillus capsici* TT41) because they showed a survival rate of up to 50% and good growth rate when treated with 30% PEG 6000. The three strains were treated with kimchi cabbage to confirm their enhanced drought stress resistance under non-watering conditions. Among the three strains, the TT41 treated group showed a significant increase in various plant parameters compared with the negative control on the 7th day. We performed extensive profiling of primary and secondary metabolites from kimchi cabbage and the TT41 strain. Multivariate and pathway analyses revealed that only the TT41 group clustered with the well-watered group and showed almost the same metabolome on the 7th day. When treated with TT41, lactic acid was identified as an indicator metabolite that significantly improved drought stress tolerance. Furthermore, lactic acid treatment effectively induced drought stress tolerance in kimchi cabbage, similar to that achieved with the TT41 strain.

## 1. Introduction

Weather patterns associated with climate change, such as droughts, floods, and extreme temperatures, have increased over the past decades [1]. These climate events are expected to cause significant crop losses. This situation poses a threat to global food security and the growing food demand. Therefore, strategies to improve food tolerance in abiotic environments are essential. Various innovative breeding and biotechnological approaches have been developed to improve plant abiotic stress tolerance.

Plant root-associated microorganisms can positively influence plant tolerance to abiotic stresses [2]. It is well-established that diverse bacteria and mycorrhizal fungi can augment stress tolerance in numerous plants [3]. Various species of bacteria and fungi have been reported to promote the growth and productivity of plants and to control plant pathogens under laboratory conditions [4]. Plant growth-promoting *Rhizobacteria* (PGPRs) are soil bacteria that directly or indirectly promote plant rooting and growth of plants [5]. PGPRs influence plant growth through the fixation of atmospheric nitrogen, solubilization of insoluble phosphates, secretion of phytohormones such as indole-3-acetic acid (IAA) and gibberellin (GAs), kinetics, and production of exopolysaccharides. However, when PGPRs are used in fields exposed to natural environmental conditions, they are highly ineffective [6]. Therefore, it is becoming essential to explore the potential of PGPRs, and research is shifting to the isolation and application of natural compounds as biocontrol agents (PGPRs) unaffected by different environments [7].

Comprehensive information on the biochemical interactions between plants and microorganisms in the rhizosphere is lacking [8]. Furthermore, the mechanism of PGPR effects in plants at the metabolomic level is yet to be fully elucidated. Research on chemical communication in the rhizosphere is currently underway. In addition, these metabolomics studies are crucial for integrating transcriptomics and proteomics in systems biology to explain complex post-genomic molecular interactions and confirm the data obtained in previous genomic analyses. In a recent study, analysis of sorghum bicolor treated with *Paenivacillus alvei* showed that 49 metabolites, including amino acids, lipids, flavonoids, and phytohormones, changed in response to fungal infection [9]. In addition, several studies on PGPR-induced reprogramming of plant metabolites, such as soybean, tomato, chickpea, and maize, with different PGPR treatments have been reported [10,11,12,13,14,15]. Analyzing the metabolites produced and secreted by PGPRs in the rhizosphere, it is important to understanding plant–microbe and microbe–microbe interactions through metabolomic profiling. These include primary (amino acids, organic acids, sugars, and lipids) and secondary (vitamins, phenolics, and terpenoids) metabolites that reduce biotic and abiotic stresses and promote plant development. However, the origin of the metabolites in the rhizosphere has not been completely identified or quantified. Therefore, identifying the metabolome of rhizosphere microbes can help understand how the rhizosphere metabolite pool influences plant–microbe interactions.

This study aimed to identify the microorganisms that confer enhanced resistance to drought stress among the various abiotic stresses that plants may encounter. The effects of these microorganisms were validated, and comprehensive metabolite profiles were analyzed to investigate the impact of microorganisms and drought stress on plants. In addition, we explored the metabolite profiles of the microorganisms to understand plant–microbe interactions. Our goal was to identify the biocontrol compounds isolated from microorganisms that promote drought tolerance in *Brassica rapa* L. subsp. *pekinensis*, which was unaffected by different environments.

## 2. Materials and Methods

### 2.1. Bacterial Strains and Culture Conditions

The soil samples were collected near the Hyogal Reservoir (N 36°28′55, E 128°16′14) in Yecheon County, Gyeongsangbuk-do, South Korea, from around the roots of native plants growing in the area. Bacterial strains were isolated from riverside soil samples in tryptic soy agar (TSA) medium at 25 °C using the standard procedures described by Fred and Waksman [16]. The isolates were purified by repeated streaking. Single isolates were stored at −70 °C in a 20% glycerol solution. Isolates were identified by comparing their 16S rRNA gene sequences to those in EzBioCloud [17].

### 2.2. Drought Tolerance Screening of Isolates

To assess the drought stress resistance of the isolated bacterial strains, we induced artificial drought stress in cells using polyethylene glycol 6000 (PEG 6000) [3]. PEG 6000 was added to the Luria–Berani (LB) broth medium at concentrations of 10, 20, and 30%. To determine whether the strains used PEG 6000 as an energy source, they were cultured in an M9 medium supplemented with 20% PEG 6000. The cultures were incubated at 180 rpm for three days. During the incubation period, growth curves were determined by measuring the absorbance at a wavelength of 600 nm with a UV–Vis spectrophotometer (UV-1800, Shimadzu, Tokyo, Japan).

### 2.3. Plant Growth Conditions and Treatments

The greenhouse experiment was conducted during the summer of 2022. Glass greenhouses with digitally controlled sprinklers were used to grow plants and conduct the experiments. Kimchi cabbage seeds (*Brassica rapa* L. subsp. *pekinensis*) “Bulam No. 3” were purchased from Hungnong Seed Co. Ltd. (Pyeongtaek, Korea). Three sets of plates, with five pots per plate, were used to obtain a minimum of 15 replicates per treatment. The bacterial cells were suspended in sterile tap water at 1 × 10^7^ CFU/mL and irrigated around the plant roots at a density of 20 mL/pot. The control group was treated with an equal volume of sterile tap water instead of bacterial suspension. The treatments were applied three times at intervals of 7 days, starting at the 4-leaf phase of the cabbage, with a final treatment at the 8-leaf phase (21, 28, and 35 days after seeding). After the final treatment on day 35, watering was stopped, and the positive control was watered twice daily. To determine the recovery rate, the plants were watered twice daily for 3 days, and then, after 7 days, they were without water.

### 2.4. Validation of Drought Resistance Enhancement in Plants by Lactic Acid

The greenhouse experiment was conducted during the summer of 2023, maintaining the same conditions as those of the 2022 experiments for both the greenhouse environment and plant growth. Lactic acid was diluted to concentrations of 1, 10, and 100 mM and applied to plant roots at 20 mL/pot. These treatments were administered three times at 7-day intervals. Observations were conducted for seven days following the last treatment, during which no additional watering was provided.

### 2.5. Phenotype and Endogenous Change Measurement

After watering was stopped, 4 pots of plants were randomly selected for the experiment at 0, 1, 3, 5, and 7 days. The soil was removed from the roots and the fresh weight of the plant was measured, whereas relative water content (RWD), malondialdehyde (MDA) content, total chlorophyll content, leaf length, and leaf width were measured from the largest leaves of each plant. Relative water content (RWC) was measured as described previously by Patanè et al. [18] and was calculated using the following equation:

$$RWC\left(\%\right)=\frac{w_{f}-w_{d}}{w_{t}-w_{d}},$$

where *W_f_* is fresh leaf weight, *W_t_* is turgid weight, and *W_d_* is dry leaf weight. MDA content was determined using the method described by Zhou et al. [19]. Chlorophyll was extracted as described by Qi et al. [20], and the total chlorophyll content was calculated according to Lichtenthaler’s method [21].

### 2.6. Metabolite Profiling

Metabolite profiling was performed to evaluate hydrophilic (amino acids, organic acids, sugars, and phenolic acids) and lipophilic metabolites (tocopherols, phytosterols, policosanols, carotenoids, and glucosinolates). Hydrophilic compounds (amino acids, organic acids, and sugars) were analyzed using gas chromatography-time-of-flight mass spectrometry (GC-TOF-MS; LECO, St. Joseph, MI, USA) [22]. Briefly, to extract hydrophilic compounds, 1 mL of extraction solution (methanol:water:chloroform = 2.5:1:1 (*v*/*v*/*v*)) and 60 μL of ribitol (200 μg/mL in methanol) were added to the sample tube. The samples were incubated at 37 °C for 30 min with shaking, then centrifuged at 4 °C at 16,000× *g* for 3 min. The 800 μL of the supernatant was mixed with 400 μL of water and centrifuged at 4 °C at 16,000× *g* for 3 min. After transferring 900 μL of the water/methanol supernatant to a new tube, the solvent was evaporated using a vacuum concentrator (CVE-3110, EYELA, Tokyo, Japan) and freeze dryer (MCFD8512, IlShin Bio-Base Co., Ltd., Dongducheon, Korea). Afterward, 80 μL of 2% methoxyamine in pyridine solution was added to the pellet and incubated for 90 min while shaking at 30 °C. After 90 min, 80 μL of N-methyl-N-(trimethylsilyl) trifluoroacetamide (MSTFA; Sigma, St. Louis, MO, USA) was added and incubated for 30 min while shaking at 37 °C. After completion of the derivatization process, the sample was transferred to a GC vial and analyzed using GC-TOF-MS, with Pegasus BT (LECO, St. Joseph, MI, USA) equipped with a CP-SIL 8 CB column (30 m × 0.25 mm × 0.25 μm). GC-TOF-MS conditions included maintaining the initial temperature at 80 °C for 2 min, raising the temperature to 320 °C at 15 °C/min, and maintaining it for 10 min. The injection volume, temperature, and ratio were 1 μL, 230 °C, and 1:25, respectively. The compounds were confirmed based on in-house libraries and retention time information. For quantification, the area of each component was divided by that of the internal standard (ribitol).

Extraction and determination of carotenoids from lipophilic components was performed using high-performance liquid chromatography (HPLC; 1100 series; Agilent, Massy, France) [23]. For carotenoid analysis, the sample was prepared in a 15 mL conical tube, and 3 mL of 0.1% ascorbic acid in ethanol was added. After incubating in an 85 °C water bath for 5 min, 0.12 mL of 80% potassium hydroxide was added. Then, the sample was incubated in an 85 °C water bath for 10 min and cooled in ice for 5 min. For quantification, 100 μL of trans-β-Apo-8’-carotenal (25 μg/mL in ethanol) was added as an internal standard. Then, 1.5 mL of water, 0.75 mL of toluene, and 0.75 mL of hexane were added to the tube and centrifuge at 1200× *g* for 5 min. After transferring the toluene/hexane supernatant to a new tube, carotenoids were re-extracted by adding 0.75 mL of toluene and 0.75 mL of hexane to the original tube. The mixture was centrifuged at 1200× *g* for 5 min, and the supernatant was collected. A total of 3 mL of the toluene/hexane layer was purged with N_2_ gas to evaporate the solvent and then re-dissolved in 0.25 mL of dichloromethane:methanol = 1:1 (*v*/*v*) solution. Afterwards, it was filtered with a 0.5 μm syringe filter and analyzed by HPLC. Carotenoid content was determined and identified using a previously described method [23].

Glucosinolate was extracted and determined using HPLC (e2695; Waters, Milford, MA, USA) with photodiode array detector (2998; Waters, Milford, MA, USA). The sample (100 mg) was placed into a 2 mL tube, and then 1.5 mL of heated 70% methanol was added. The sample was placed in a 69 °C water bath for 5 min and centrifuged at 13,000× *g* for 10 min. After transferring the supernatant to a new tube, the process was repeated twice to collect 4.5 mL of the supernatant, which was passed through a column filled with DEAE Sephadex A-25 (GE Healthcare, Uppsala, Sweden). After washing with 3 mL of water, 75 μL of arylsulfatase solution (23.3 mg/mL) was added and reacted at room temperature for 16 h. After elution with 2.4 mL of water, it was passed through a 0.2 μm hydrophobic filter (13JP020AN, ADVANTEC, Tokyo, Japan) and analyzed by HPLC. Glucosinolates were determined and identified using a previously described method [24].

Other lipophilic compounds (policosanols, phytosterols, and tocopherols) were analyzed using GC-quadrupole (q) spectrometry [25]. To the sample, 3 mL of 0.1% ascorbic acid in ethanol and 0.05 mL of 5α-cholestane (10 μg/mL in hexane) were added as an internal standard. After incubating in an 85 °C water bath for 5 min, 0.12 mL of 80% potassium hydroxide was added. Then, the sample was incubated in an 85 °C water bath for 10 min and cooled in ice for 5 min. Then, 1.5 mL of water, 0.75 mL of toluene, and 0.75 mL of hexane were added to the tube and centrifuged at 1200× *g* for 5 min. The toluene/hexane supernatant was transferred to a new tube and re-extracted by adding 0.75 mL of toluene and 0.75 mL of hexane to the original tube. The sample was centrifuged at 1200× *g* for 5 min, and the supernatant was transferred. Then, 3 mL of the toluene/hexane layer was purged with N_2_ gas to remove all solvents. Then, 30 μL of pyridine and 30 μL of MSTFA were added and incubated at 60 °C for 30 min. The samples were transferred to a GC vial and analyzed using GC-qMS. GC-qMS was performed using Shimadzu GCMS-QP2010 (Shimadzu, Kyoto, Japan) equipped with an Rtx-5MS column (30 m × 0.25 mm × 0.25 μm). The GC-qMS oven was initially set at 80 °C for 2 min, then raised to 320 °C at 15 °C per minute and maintained at the final temperature for 10 min. The injection volume, temperature, and ratio were 1 μL, 290 °C, and 1:10, respectively. Compounds were identified based on in-house libraries and retention time information. For quantification, the content of each compound was calculated using calibration curve equations as previously described [25].

### 2.7. Statistical Analysis

All analyses were performed in triplicate. All the acquired data were normalized for principal component analysis (PCA) and orthogonal partial least squares discriminant analysis (OPLS-DA) using unit variance scaling. PCA and OPLS-DA were performed using SIMCA 13 software (Umetrics, Umeå, Sweden). The Asparagus metabolic pathway was visualized using PathVisio (version 3.3.0) [26,27]. To link the metabolites to the schematics of the metabolic pathways in PathVisio, all metabolite names were converted to PubChem compound IDs (PubChem CIDs) (Table S1). The metabolic profile data was shown using unit variance scaling from −1.0 to 1.0. A scaling value >0 is displayed in red and denotes levels higher than the average. A scaling value of <0 is displayed in green and denotes lower levels than the average. Gray color represents substances that have not yet been identified.

## 3. Results and Discussion

### 3.1. Selection of Drought Tolerance Microbial Resource

**Figure 1:**
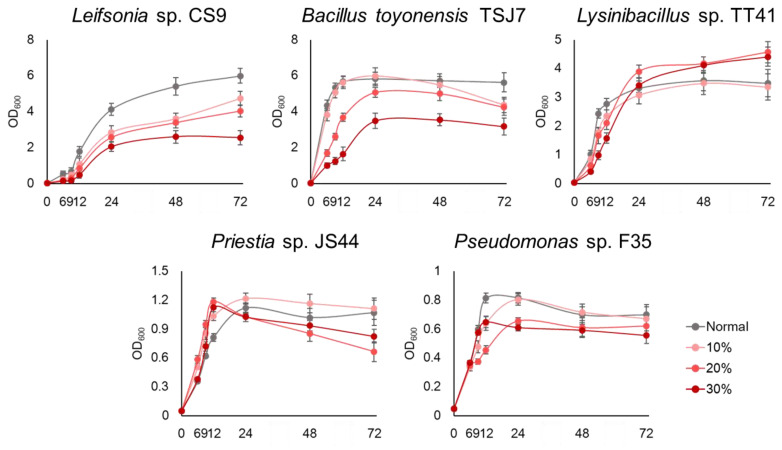
Growth curves of the isolates under drought stress. Data are expressed as the average ± STDEV values of three biological replicates per condition.

For drought tolerant strain screening, the five isolated strains (*Bacillus toyonensis* TSJ7, *Leifsonia* sp. CS9, *Lysinibacillus capsici* TT41, *Priestia* sp. JS44, and *Pseudomonas* sp. F35) were visually selected from the isolates that showed growth in 5 mL LB media with 10% PEG. These five strains were cultivated in LB media containing 10, 20, and 30% polyethylene glycol 6000 (PEG 6000) for 72 h, which was used to create a non-aqueous environment (Figure 1). All five experimental strains did not use PEG 6000 as an energy source; instead, PEG 6000 was used solely to create an osmotic condition. The five strains showed survival rates of up to 50% compared to the control (0% PEG 6000) at 30% PEG 6000 condition. Among them, TT41 showed a survival rate higher than that of the control or 10, 20, and 30% PEG 6000 conditions. The JS44 and F35 showed low growth rates (OD_600_ value less than 1 after 72 h). In contrast, TT41, CS9, and TSJ7 showed at least twice the growth rates of JS44 and F35 after 72 h in 30% PEG 6000 condition.

Therefore, we selected TT41, CS9, and TSJ7 as the putative strains affecting plant drought stress tolerance.

### 3.2. Plant Drought Tolerance Effect of the Microbial Resource

**Figure 2:**
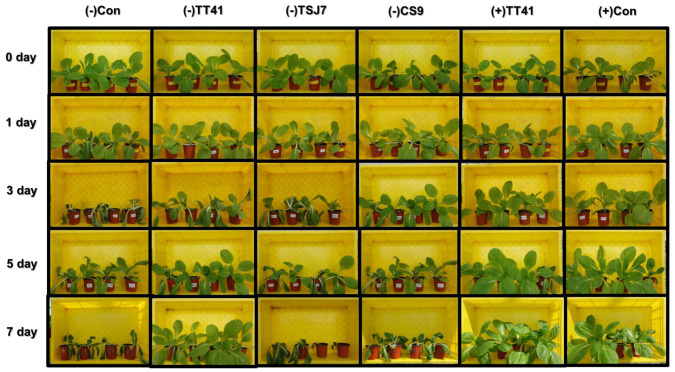
Effects of three selected strains on kimchi cabbage (*Brassica rapa* L. subsp. *pekinensis*) under control and drought conditions. Images were taken at 0, 1, 3, 5, and 7 days after watering was stopped. (−)Con: drought treated negative control, (−)TT41: *Lysinibacillus* sp. TT41 inoculated and drought treated, (−)CS9 *Leifsonia* sp. CS9 inoculated and drought treated, and (−)TSJ7: *Bacillus toyonensis* TSJ7 inoculated and drought treated, (+)TT41 *Lysinibacillus* sp. TT41 inoculated and well-watered treated, (+)Con: well-watered positive control.

To confirm the effect on plant drought tolerance, an experiment was performed using kimchi cabbage (*Brassica rapa* L. subsp. *pekinensis*) treated with three selected strains (*Lysinibacillus capsici* TT41, *Leifsonia* sp. CS9 and *Bacillus toyonensis* TSJ7). Six experimental groups were designed: (1) (+)Con: a well-watered positive control, (2) (−)Con: a drought treated negative control, (3) (−)TT41: a TT41 strain inoculated and drought treated, (4) (+)TT41: a TT41 strain inoculated and well-watered, (5) (−)CS9: a CS9 strain inoculated and drought treated, and (6) (−)TSJ7: a TSJ7 strain inoculated and drought treated (Figure 2). In addition, the drought treated groups were incubated for 7 days after irrigation was stopped. All six groups were harvested on days 0, 1, 3, 5, and 7. Under drought stress, the (−)Con, (−)CS9, and (−)TSJ7 groups withered after 7 days of cultivation (Figure 2). In contrast, the (−)TT41 group grew well under drought stress. However, the (−)TT41 was showed lower growth rate than well-watered groups((+)Con and (+)TT41). On day 7, under drought stress, a decrease in (−)Con leaf number (46.15%), leaf length (14.04%), leaf width (30.52%), fresh weight (77.93%), leaf relative water content (52.43%), and chlorophyll content (24.56%) were observed compared to those of (+)Con (Table 1). In addition, the malondialdehyde (MDA) content, which is an indicator of oxidative damage in the leaf tissues, increased by 45%. In contrast, the (−)TT41 induced a significant increase in plant leaf number (25.05%), leaf length (5.42%), leaf width (13.44%), fresh weight (47.88%), leaf relative water content (47.53%), and chlorophyll content (25.05%) under drought stress compared with (−)Con. Furthermore, (−)TT41 caused a 32.27% decrease in MDA content. (−)TSJ7 and (−)CS9 were induced to also increase the leaf number (28.20 and 19.26%), leaf length (6.22 and -5.53%), leaf width (14.13 and 8.00%), fresh weight (39.60 and 26.72%), and chlorophyll content (35.21 and 29.72%) in drought condition compared to (−)Con. However, (−)TSJ7 did not significantly affect leaf relative water or MDA content. The (−)CS9 induced a significant decrease in MDA content, but there was little significant increase in leaf relative water content compared with (−)Con. Under well-watered conditions, the isolated TT41 strain induced approximately a 10% increase in leaf relative water and chlorophyll content compared with (+)Con. The results showed that(−) TT41 had almost the same plant parameters as (+)Con and (+)TT41 on day 7. In contrast, (−)TSJ7 and (−)CS9 withered after 7 days of cultivation, similar to (−)Con. Therefore, we demonstrate that TT41 regulates drought stress tolerance in kimchi cabbage.

### 3.3. Effect of Microbial Resource on Plants’ Endogenous Metabolome

To confirm the metabolomic responses to drought stress, we performed comprehensive metabolite profiling of kimchi cabbage using GC-TOF-MS, GC-qMS, and HPLC. We detected 50 hydrophilic metabolites, including amino acids, organic acids, sugars, and phenolic acids (Table S2). Furthermore, 21 lipophilic compounds, including three tocopherols, three phytosterols, nine policosanols, six carotenoids, and three glucosinolates, were identified in kimchi cabbage (Tables S3 and S4).

Multivariate statistical analyses are useful for extracting information from complex experimental datasets. Metabolite profile data were subjected to principal component analysis (PCA). PCA converts the original data into a new set of variables, called principal components (PCs), using an orthogonal linear transformation. Then, the scores and loadings of the PCs were expressed as a bi-dimensional plot. These plots (score and loading plots) represent data patterns from the samples. The data were normalized using unit variance scaling.

The PCA results showed a good separation in PC 1 according to the period when irrigation was stopped (Figure S1). Two PCs of the score plots explained 51.8% of the total variance (PC1: 33.6%; PC2: 18.2%). The kimchi cabbages group 0 and 1 days after irrigation stopped were clearly separated from the group 3–7 days of drought stress. These results indicate that drought stress had an effect on metabolomics profile after three days. In addition, the separation of the samples changed from positive to negative PC1 over time. In the group of samples after 3–7 days of watering pause, PC2 contributed to the separation according to the degree of drought stress. The well-watered (no drought stress: (+)Con and (+)TT41) samples were grouped with (−)TT41, and this group was located on negative values of PC2. The drought stressed samples ((−)Con, (−)TSJ7, and (−)CS9), except for (−)TT41, were clustered and located on the positive PC2 at 7 days. After 3 days, the (−)TSJ7 and (−)CS9 were clustered with the well-watered groups. However, after 5 days, (−)TSJ7 moved to the drought stressed group. After 7 days, (−)TSJ7 and (−)CS9 clustered with the drought stressed group, except for (−)TT41. These results indicated that the TT41 strain effectively reduced drought stress in kimchi cabbages. The strains TSJ7 and CS9 affected the drought stress tolerance of the cabbage samples for a short period.

**Figure 3:**
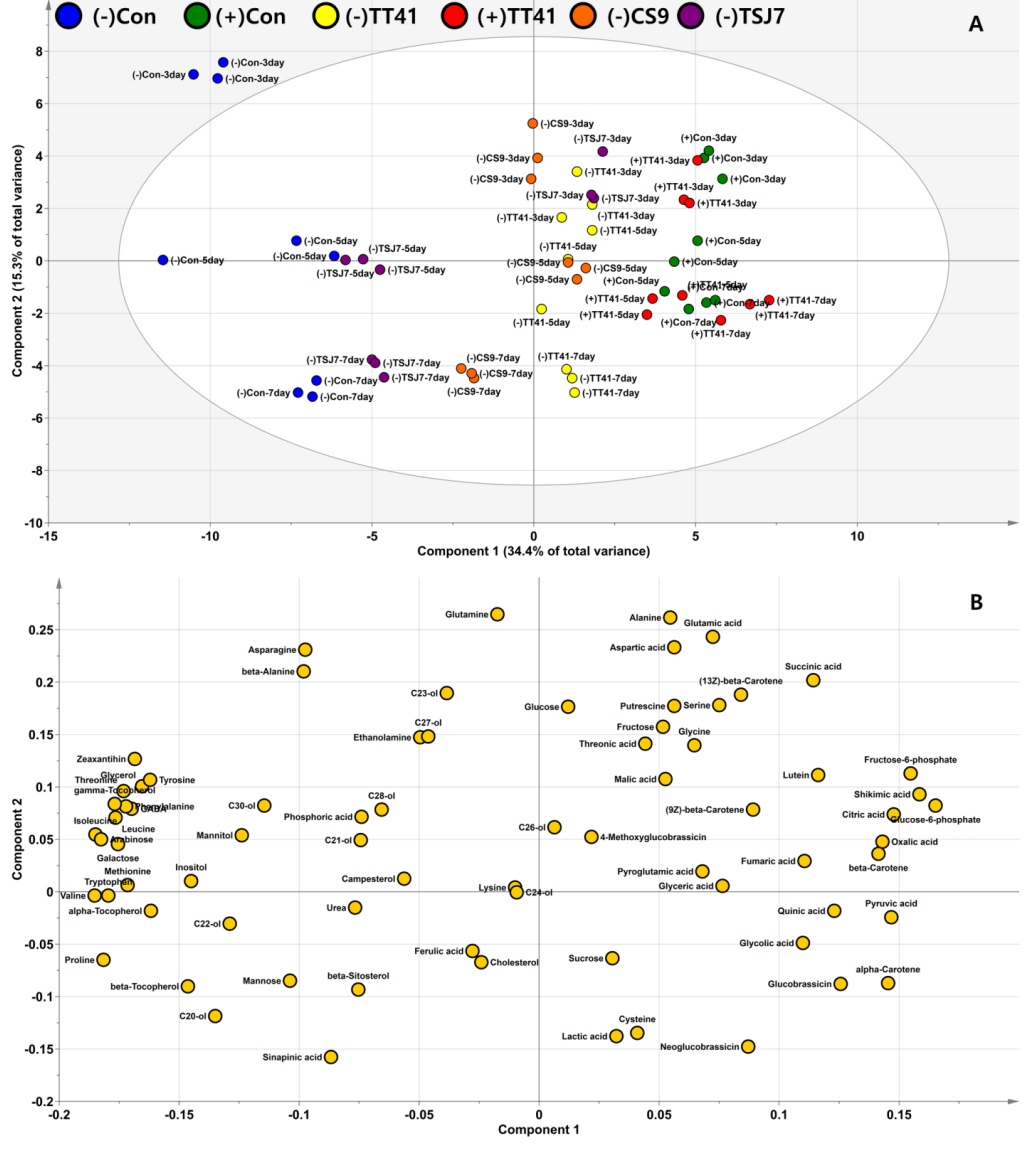
Principal component analysis (PCA), score plots (**A**), and loading plots (**B**) of selected three strains of treated kimchi cabbage (*Brassica rapa* L. subsp. *pekinensis*) under well-water or drought conditions. Group configuration: (+)Con: well-watered positive control, (−)Con: drought treated negative control, (−)TT41: TT41 strain inoculated and drought treated, (+)TT41: TT41 strain inoculated and well-watered treated, (−)CS9: CS9 strain inoculated and drought treated, and (−)TSJ7: TSJ7 strain inoculated and drought treated.

The duration of stress and treatment with microorganisms caused shifts in the metabolomic profile and influenced the peculiarities of grouping cabbage samples after 3–7 days of drought stress (Figure 3). The PCA results showed a good separation between PC1 (34.4%; drought stress) and PC2 (15.3%; period of watering cessation). Score plots showed a clearer separation of samples at 3–7 days compared with the score plot in Figure S1 (Figure 3A). In the loading plot of component 1, glycolysis, TCA cycle-related metabolites (organic acids), and carotenoids had significantly positive eigenvector values (Table S5). In contrast, the significantly negative eigenvector values of the metabolites in PC1 were tocopherols, polyols, and amino acids. To adapt to drought stress, plants synthesize essential protective compounds (osmolytes) for osmotic regulation [1]. The accumulation of osmolytes in the cell maintains water potential or stimulates water uptake. Common osmolytes include sugars, polyols, amino acids, and acid derivatives [1]. In this study, drought stressed samples ((−)Con, (−)TSJ7, and (−)CS9), except for (−)TT41, showed high levels of hydrophobic amino acids (alanine, valine, leucine, isoleucine, methionine, phenylalanine, tyrosine, tryptophan), proline, GABA, mannitol, inositol, and tocopherols (Figure 3B). Previously, it was reported that osmotic stress-induced proteins are crucial in the adaptation of plants to water deficit and are enriched in hydrophobic amino acids [28]. These amino acids are essential for the structural stabilization of osmotic stress-induced proteins through hydrophobic interactions. Proline is one of the most important indicators of stress in plants, and it has been reported that plants overaccumulate proline to improve their tolerance to abiotic stress [1]. It has been suggested that GABA plays a central role in the TCA cycle [1]. Stimulation of GABA in response to the increased demand for TCA cycle intermediates has been shown to support the metabolism of secondary metabolites for plant survival under drought stress conditions. Polyols, such as mannitol and inositol, are recognized to be linked to protection against dehydration and act to slow the rate of water loss during desiccation [1]. Tocopherols are well-known nonenzymatic antioxidants. Previous studies have reported that tocopherols are significantly upregulated under drought stress [29]. In contrast, the well-watered samples ((+)Con and (+)TT41) showed high energy metabolism and carotenoid content for plant development and growth. Significant metabolites in PC2 were glutamine, alanine, glutamic acid, aspartic acid, cysteine, lactic acid, sinapinic acid, and neoglucobrassicin. The samples under short-term drought stress conditions (0–3 days) showed high levels of glutamine, alanine, glutamic acid, and aspartic acid and a significant positive contribution to PC2 (Figure 3B). In contrast, low levels of these amino acids were found in the samples under drought stress conditions for 7 days. A previous study reported that glutamine, alanine, glutamic acid, and aspartic acid content decreased under drought stress conditions [30]. Lactic acid, cysteine, and glucosinolates showed significant negative contributions to PC2 and high levels in isolated strain-treated samples ((−)TSJ7, (−)CS9, and (−)TT41) after 7 days of drought stress. Li et al. reported that cysteine levels increased when white clover was treated with beta-sitosterol, a plant growth regulator, to induce tolerance to water stress under drought stress [31]. Furthermore, beta-sitosterol treatment increased lactic acid, glycolic acid, glyceric acid, shikimic acid, and quinic acid levels under drought stress, but did not induce intermediates of the TCA cycle. These increased metabolites have been reported to influence plant growth and regulate osmotic and redox balance under drought stress. In this study, metabolites (lactic acid, cysteine, glycolic acid, glyceric acid, shikimic acid, and quinic acid) significantly contributed to TT41 strain treated kimchi cabbage. These results indicated that TT41 regulates drought stress tolerance in kimchi cabbage. Glucosinolates increased over time and only in the TT41 treatment under water-deficient conditions.

**Figure 4:**
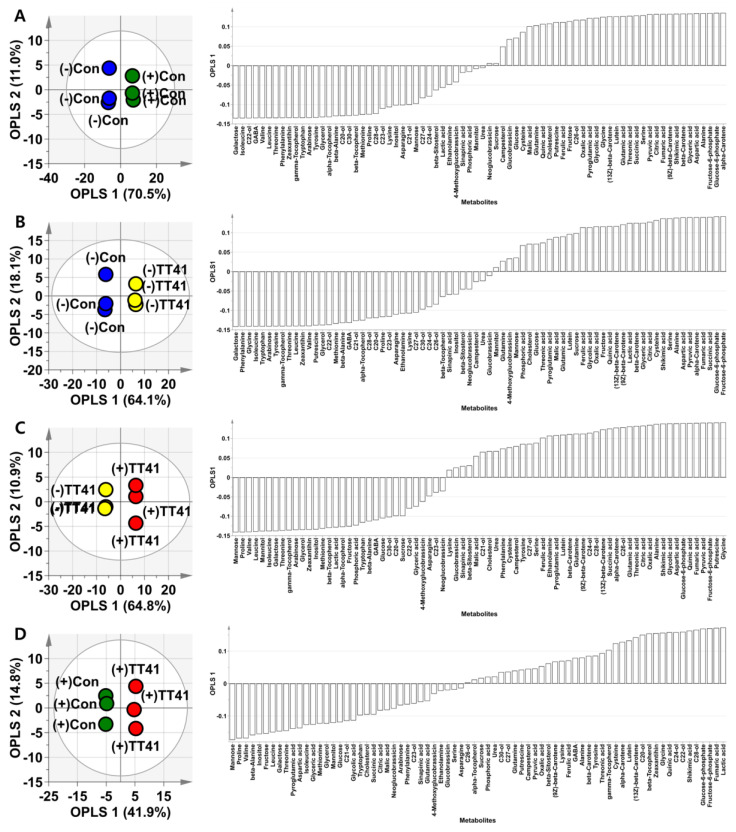
OPLS-DA score plots and loading column plots of TT41 strain treated kimchi cabbage (*Brassica rapa* L. subsp. *pekinensis*) under well-water or drought conditions. The groups consisted of comparing (−)Con and (+)Con (**A**), (−)Con and (−)TT41 (**B**), (−)TT41 and (+)TT41 (**C**), and (+)Con and (+)TT41 (**D**). Group configuration: (+)Con: well-watered positive control, (−)Con: drought treated negative control, (−)TT41: TT41 strain inoculated and drought treated, and (+)TT41: TT41 strain inoculated and well-watered treated.

To identify significant metabolites that induce drought stress tolerance in the TT41 strain, OPLS-DA was performed with four groups wherein a combination of (−)Con, (+)Con, (−)TT41, and (+)TT41 group of cabbage samples was used (Figure 4 and Table S6). Four groups of samples were compared A: (−)Can and (+)Can; B: (−)Can and (−)TT 41, C: (−)TT41 and (+)TT41; D: (+)Can and (+)TT41. When comparing groups A and B, the patterns of the contributing metabolites were almost identical, except that lactic acid and cysteine contributed only to (−)TT41 (Figure 4A,B). In group C, (−)TT41 and (+)TT41 were shown to differ in metabolites produced by drought stress. Lactic acid was found to be a significant metabolite contributor to (−)TT41, but cysteine was not a significant contributor (Figure 4C). Furthermore, the OPLS-DA results for group D showed metabolite differences following TT41 treatment under well-watered conditions (Figure 4D). Lactic acid was the most important contributor to (+)TT41, and cysteine was also a significant contributor. These OPLS-DA results indicated that lactic acid and cysteine could induce drought stress tolerance in kimchi cabbage treated with the TT41 strain.

### 3.4. Metabolite Profiling of Lysinibacillus sp. TT41 Strain under Drought Stress

**Figure 5:**
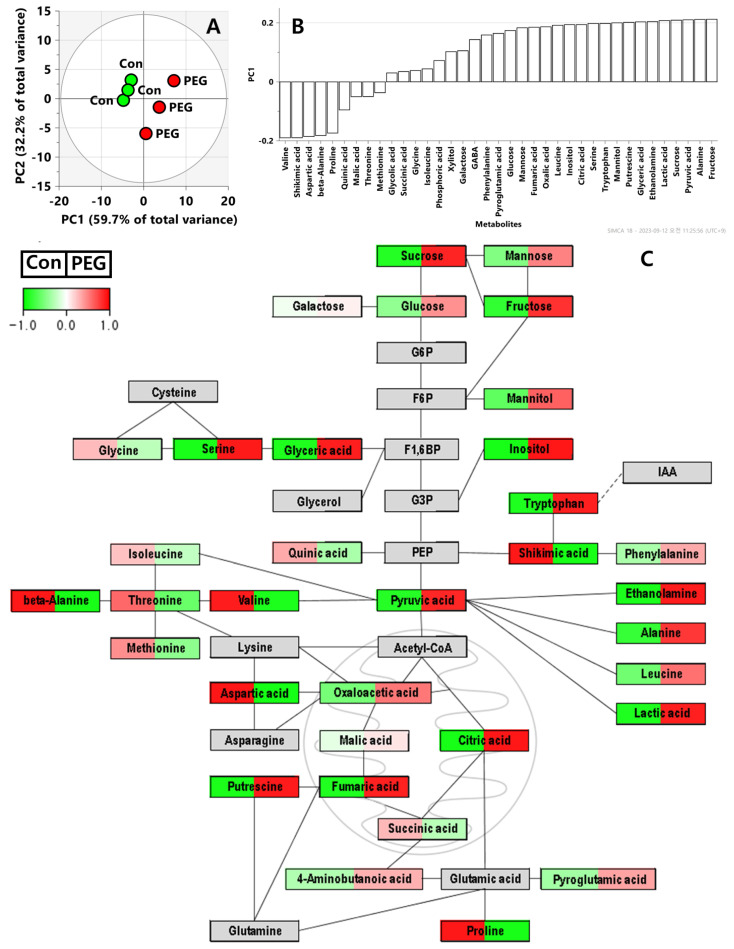
PCA score plots (**A**) and loading column plots (**B**) of TT41 strain (control; Con and 30% of PEG treatment; PEG). Metabolic pathway diagrams of TT41 strain (control; Con and 30% of PEG treatment; PEG) visualized by PathVisio (**C**). Unit variance(UV) scaling range is from −1.0 to 1.0. A scaling value >0 is displayed in red and denotes levels higher than the average. A scaling value of <0 is displayed in green and denotes lower levels than the average. Gray color represents substances that have not yet been identified.

We successfully demonstrated that the TT41 strain induces drought stress tolerance in kimchi cabbage. Additionally, when treated with TT41, lactic acid and cysteine were identified as putative indicators of drought stress tolerance in kimchi cabbage. Therefore, to reveal the metabolic co-effects of kimchi cabbage and TT41 strain, we performed metabolite profiling of the TT41 strain (Control and 30% of PEG 6000 treatment) using GC-TOF-MS. We detected 37 metabolites, including amino acids, organic acids, and sugars (Table S7). The metabolite profiling results were subjected to PCA. The PCA results showed a good separation between Con and PEG in PC 1 (Figure 5A). In the loading plot, the TT41 strain subjected to PEG 6000 treatment showed a positive PC1 value (Table S8). In addition, most metabolites, including glycolysis-and TCA cycle-related metabolites, as well as amino acids, except valine, shikimic acid, aspartic acid, beta-alanine, and proline, contributed positively to PC1 (Figure 5B).

*Lysinibacillus* spp. are valuable plant growth promoters with high indole-3-acid (IAA) production [32]. IAA is a plant hormone belonging to the auxin class, and its biosynthetic pathway begins with tryptophan [33]. A previous study detected genes associated with tryptophan and IAA synthesis and metabolism in 12 *Lysinibacillus* spp. [32]. In this study, tryptophan levels were high in *Lysinibacillus* sp. TT41 (Figure 5C). Thus, in the TT41 strain, we expected the synthesis of IAA to be upregulated by high levels of tryptophan. However, in the present study, under well-watered conditions, the growth rate of the TT41 strain treated kimchi cabbage was similar to that of (+)Con (Figure 2 and Table 1). Thus, the TT41 strain had a low plant growth-promoting capacity, which may be due to its low metabolism for IAA synthesis.

As shown in Figure 5, most energy metabolism-related metabolites and amino acids significantly contributed to PEG in PC1. These results are consistent with the growth curve of the TT41 strain after PEG 6000 treatment (Figure 1). The PEG 6000-treated TT41 strain exhibited a higher growth rate than the control. Based on these results, the TT41 strain might show high energy and amino acid metabolism for growing under drought–stress conditions. Among these metabolites, lactic acid showed high positive eigenvector values (0.2079) in PC1 and was the most significant contributor in TT41 strain-treated kimchi cabbage (Figure 5B and Table S8). A previous study reported that treatment with *Lysinibacillus fusiformis* L. (PLT16) mitigated drought stress in soybean plants [34]. In addition, lactic, malic, and citric acid levels increased in soybean plants treated with PLT16 under drought stress. The increase in lactic acid in both the TT41 strain and treated plants suggests that lactic acid secretion by the TT41 strain may be a key metabolic pathway for reducing drought stress in plants (Figure 5C).

### 3.5. Effect of Lactic Acid Treatment on Kimchi Cabbage under Drought Stress

Based on the results of the afore mentioned experiment, lactic acid was identified as a putative drought stress-reducing metabolite. Therefore, kimchi cabbage was treated with lactic acid under drought stress. Lactic acid (100, 10, 1 mM) and TT41 strain treatments were applied three times at an interval of 7 days (21, 28, and 35 days after seeding) before watering stopped.

**Figure 6:**
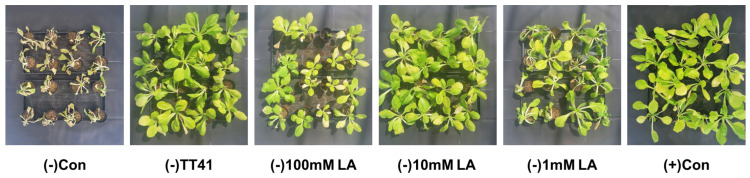
Effects of lactic acid (LA) and TT41 on kimchi cabbage (*Brassica rapa* L. subsp. *pekinensis*) under drought stress.

**Figure 7:**
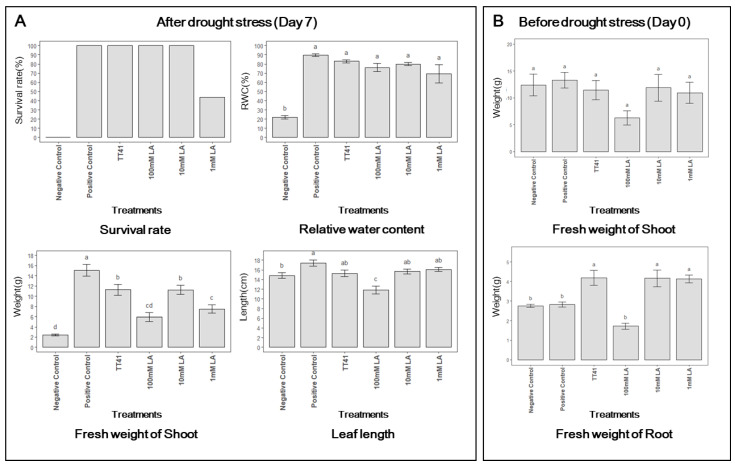
Effects of lactic acid (LA) and TT41 on kimchi cabbage (*Brassica rapa* L. subsp. *pekinensis*) under drought stress. The results measured after 7 days of drought condition (**A**) and those obtained before the drought condition (**B**). Data represent mean ± SE; *n* = 16 pot per treatment. Different letters indicate significant differences between the treatments (Tukey test, *p* < 0.05).

Treatment with 10 mM lactic acid resulted in almost the same phenotypes as the TT41 strain treatment after 7 d of watering pause (Figure 6). Compared to the 10 mM lactic acid treatment, the 100 mM lactic acid treatment resulted in high survival and maintenance of the relative water content, but growth was inhibited. In the 1 mM Lactic acid treatment group, more than half of the plants died due to drought stress, and the effect on the maintenance of relative water content was lower than that in the other treatments. The survival rate after 7 days was 100% in (+)Con, (−)TT41, (−)10mM LA, and (−)100 mM LA (Figure 7A). However, (−)1 mM LA showed a survival rate of 43.75%, and (−)Con was completely wilted. The leaf length of (+)Con was the longest, while (−)100 mM LA was the shortest. In addition, (−)Con, (−)TT41, 1 mM LA, and 10 mM LA showed almost similar leaf lengths. Compared to (+)Con, the fresh weights of (−)TT41 and (−)10 mM LA were reduced by 25.2 and 25.3%, respectively. However, (−)1 mM LA, (−)100 mM LA, and (−)Con showed fresh weight reductions of 50.4, 60.8, and 83.8%, respectively. All treated samples showed almost the same relative water content as (+)Con. The results showed that lactic acid was effective in inducing drought stress tolerance in kimchi cabbage, similar to that of the TT41 strain. In addition, on day 0 (before the watering phase), the fresh weights of all cabbages were similar, except for the (−)100 mM LA (Figure 7B). In contrast, the fresh weight of the root increased only in (−)TT41, (−)1 mM LA, and (−)10 mM LA. These results indicate that the TT41 strain and lactic acid promoted root growth.

Bacteria produce lactic acid as a by-product of metabolic activity [35]. According to Chen et al. [36], a low dose of L-lactic acid could significantly affect diverse aspects of plant growth and soil characteristics. L-lactic acid was beneficial for plant growth, primarily by enhancing plant photosynthesis, influencing rhizosphere microorganisms and element utilization, and increasing tolerance to osmotic stress. In addition, d-lactic acid secreted by microorganisms elicits pattern-triggered immunity (PTI) priming in plants. PTI signaling triggers plant defense mechanisms such as stomatal closure, enabling plants to acquire resistance to abiotic stress [37]. Therefore, we confirmed a strong correlation between the increased lactic acid in bacterial metabolomes under drought–stress conditions and the increased lactic acid in plant metabolomes due to TT41 strain treatment, demonstrating their significant association with the promotion of drought stress in kimchi cabbage. This supports the hypothesis that an appropriate concentration of lactic acid helps alleviate drought stress in kimchi cabbage.

## 4. Conclusions

In this study, we successfully developed a microbial biocontrol agent for drought stress in kimchi cabbage (*Brassica rapa* L. subsp. *pekinensis*). TT41 strain treatment significantly increased various plant parameters compared with the negative control under drought stress on day 7. Metabolite profiling and multivariate analyses showed that only the TT41 strain-treated kimchi cabbage had an almost identical metabolome to that of the well-watered groups under drought stress on day 7. When treated with TT41, lactic acid was identified as an indicator metabolite that significantly improved drought stress tolerance. In addition, metabolite profiling of the TT41 strain under 30% PEG 6000 conditions also showed an increase in lactic acid. Therefore, we treated kimchi cabbage with lactic acid under drought stress. The lactic acid treatment effectively induced drought stress tolerance in kimchi cabbage as the TT41 strain. These findings provide new insights for understanding the metabolite network interactions between plants and microorganisms in the rhizosphere under drought stress and should support the cultivation of kimchi cabbage under drought stress conditions.

## Figures and Tables

**Table 1:** Effects of selected strains on kimchi cabbage (*Brassica rapa* L. subsp. *pekinensis*) stress tolerance under control and drought treatments on the 7th day.

Treatment	Leaf Number	Leaf Length (cm)	Leaf Width (cm)	Fresh Weight (g)	Leaf Relative Water Content (%)	Chlorophyll Content (mg/g^FW^)	Malondialdehyde Content (mg/g^FW^)
(+)Con	13.00 ± 0.82 a	19.08 ± 2.35 a	9.27 ± 1.08 a	35.67 ± 9.75 a	84.7 ± 7.50 a	31.34 ± 1.20 ab	6.88 ± 1.03 ab
(−)Con	7.00 ± 0.00 c	16.40 ± 0.30 a	6.44 ± 0.21 c	7.87 ± 0.82 b	40.29 ± 2.83 d	23.64 ± 2.53 b	10.04 ± 0.64 ab
(−)TT41	9.34 ± 1.25 bc	17.34 ± 1.32 a	7.44 ± 0.38 bc	15.10 ± 2.41 b	76.79 ± 7.00 ab	31.54 ± 1.73 ab	7.59 ± 1.04 ab
(+)TT41	13.00 ± 0.82 a	19.00 ± 1.19 a	8.74 ± 0.53 ab	32.37 ± 6.1 a	93.36 ± 5.76 a	35.84 ± 3.11 ab	6.28 ± 0.61 b
(−)TSJ7	9.75 ± 1.09 b	17.55 ± 0.46 a	7.50 ± 0.44 bc	13.03 ± 0.8 b	55.19 ± 11.99 cd	36.49 ± 4.19 a	10.89 ± 2.31 a
(−)CS9	8.67 ± 0.48 bc	15.54 ± 0.72 a	7.00 ± 0.46 c	10.74 ± 2.5 b	63.78 ± 5.01 bc	33.64 ± 9.99 ab	7.64 ± 0.72 ab

(+)Con: well-watered positive control, (−)Con: drought treated negative control, (−)TT41: *Lysinibacillus* sp. TT41 inoculated and drought treated, (+)TT41 *Lysinibacillus* sp. TT41 inoculated and well-watered treated, (−)CS9 *Leifsonia* sp. CS9 inoculated and drought treated, and (−)TSJ7: *Bacillus toyonensis* TSJ7 inoculated and drought treated. Each data point represents the mean of four replicates. Error bars represent standard deviation. The bars with different letters are significantly different from each other, as evaluated using Tukey’s test.

## Data Availability

The authors declare that the data supporting the findings of this study are available within the paper and its Supplementary Information Files.

## Supplementary Materials

The following supporting information can be downloaded from https://www.mdpi.com/article/10.3390/metabo14020087/s1. Figure S1: Principal component analysis (PCA) score plots (A) and loading plots (B) of selected 3 strains of kimchi cabbage (*Brassica rapa* L. subsp. *pekinensis*) under well-water or drought conditions; Table S1: Pubchem CID of metabolites and unit variance (UV) scaling transformation data of metabolites in TT41 strain; Table S2: Composition and content (ratio/g) of hydrophilic compounds in Kimchi cabbages; Table S3: Composition and content (µg/g) of terpenoid and policosanol compounds in kimchi cabbages; Table S4: Composition and content (µg/g) of glucosinolate compounds in kimchi cabbages; Table S5: Loadings of the variables in the principal components; Table S6: Loadings of the variables in the OPLSs; Table S7: Composition and content (ratio/g) of hydrophilic compounds in TT41 strain; Table S8: Loadings of the variables in the principal components.
