# Supplementary material for: Metabolomics Reveals Lysinibacillus capsici TT41-Induced Metabolic Shifts Enhancing Drought Stress Tolerance in Kimchi Cabbage (Brassica rapa L. subsp. pekinensis)
Source: Metabolites. 2024 Jan 25;14(2):87. doi: 10.3390/metabo14020087 (PMC10890545; doi:10.3390/metabo14020087)
Supplement: Supplementary file 1 [file metabolites-14-00087-s001.zip › metabolites-2745524 - Metabolites_Supplementary data_Figure.pdf]

Supplementary Materials

Metabolomics Reveals *Lysinibacillus capsici* TT41-Induced Metabolic Shifts Enhancing Drought Stress Tolerance in Kimchi Cabbage (*Brassica rapa* L. subsp. Pekinensis)

Tae Jin Kim [1,†], Ye Ji Hwang [1,†], Young Jin Park [2], Jong Sung Lee [2], Jae Kwang Kim [2,*] and Mi-Hwa Lee [1,*]

[1] Bio-Resource Industrialization Center, Nakdonggang National Institute of Biological Resources, Sangju 37242, Republic of Korea

[2] Division of Life Sciences, College of Life Sciences and Bioengineering, Incheon National University, Incheon 22012, Republic of Korea

* Correspondence: kjkpj@inu.ac.kr (J.K.K.); blume96@nnibr.re.kr (M.-H.L.); Tel.: +82-32-835-8241 (J.K.K.); +82-54-530-0870 (M.-H.L.)

† These authors contributed equally to this work.

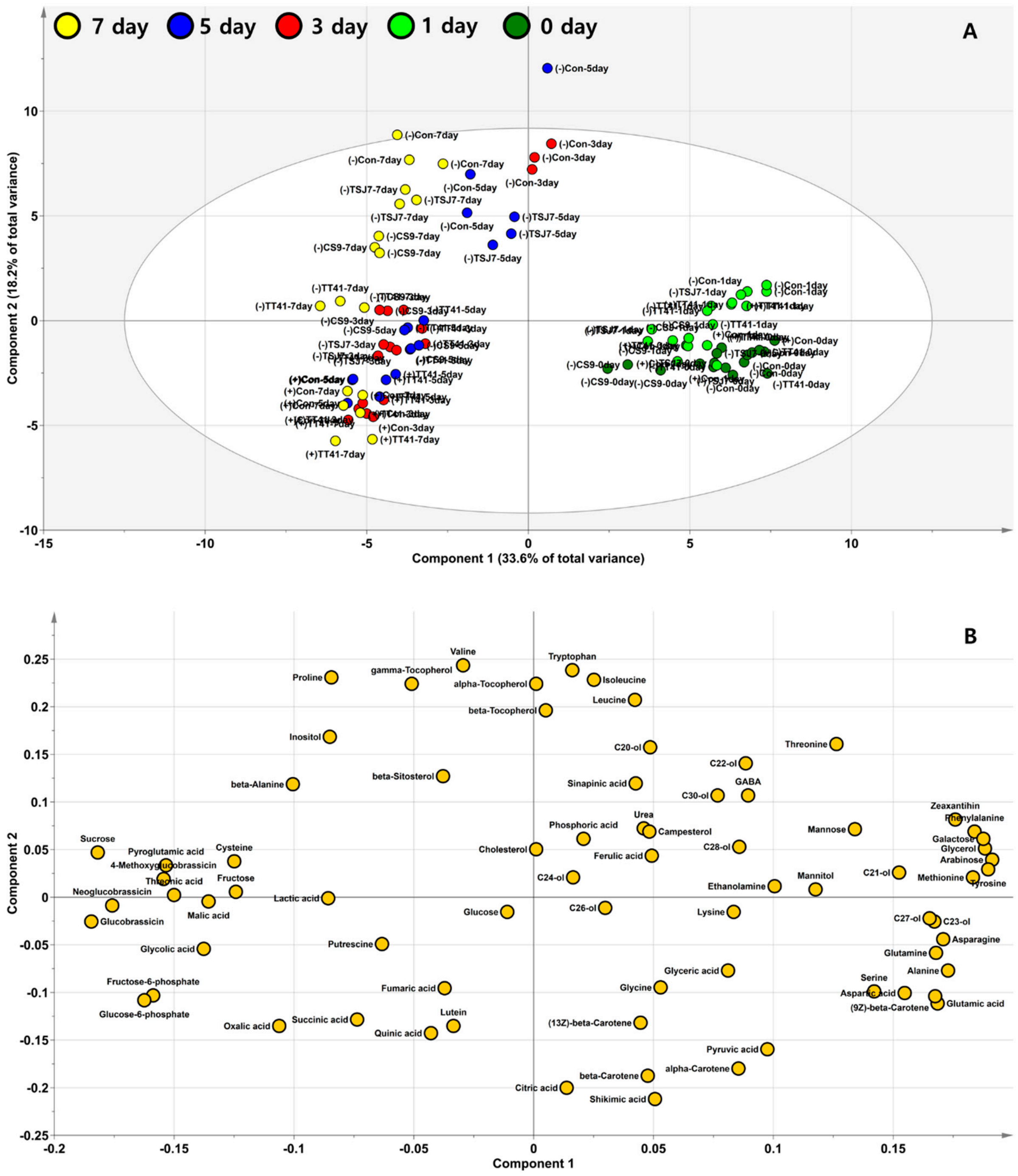

Figure S1. Principal component analysis (PCA) score plots (**A**) and loading plots (**B**) of selected 3 strains treated kimchi cabbage (*Brassica rapa* L. subsp. *Pekinensis*) under well-water or drought conditions. Group configuration : (+)Con: well-watered positive control, (−)Con: drought treated negative control, (−)TT41: TT41 strain inoculated and drought treated, (+)TT41: TT41 strain inoculated and well-watered treated, (−)CS9: CS9 strain inoculated and drought treated, and (−)TSJ7: TSJ7 strain inoculated and drought treated.
